# Pulsed SILAC-based proteomic analysis unveils hypoxia- and serum starvation-induced *de novo* protein synthesis with PHD finger protein 14 (PHF14) as a hypoxia sensitive epigenetic regulator in cell cycle progression

**DOI:** 10.18632/oncotarget.26669

**Published:** 2019-03-15

**Authors:** Jung Eun Park, Shun Wilford Tse, Guo Xue, Christina Assisi, Aida Serra Maqueda, Gallart Palau Xavier Ramon, Jee Keem Low, Oi Lian Kon, Chor Yong Tay, James P. Tam, Siu Kwan Sze

**Affiliations:** ^1^ Division of Structural Biology and Biochemistry School of Biological Sciences, Nanyang Technological University, Singapore 637551; ^2^ Department of Oncology, Tan Tock Seng Hospital, Singapore 308433; ^3^ Laboratory of Applied Human Genetics, Division of Medical Sciences, National Cancer Centre Singapore, Singapore 169610; ^4^ Division of Materials Technology School of Materials Science and Engineering, Nanyang Technological University, Singapore 639798

**Keywords:** hypoxia, pSILAC, PHF14, cell cycle inhibition

## Abstract

Hypoxia is an environmental cue that is associated with multiple tumorigenic processes such as immunosuppression, angiogenesis, cancer invasion, metastasis, drug resistance, and poor clinical outcomes. When facing hypoxic stress, cells initiate several adaptive responses such as cell cycle arrest to reduce excessive oxygen consumption and co-activation of oncogenic factors. In order to identify the critical novel proteins for hypoxia responses, we used pulsed-SILAC method to trace the active cellular translation events in A431 cells. Proteomic discovery data and biochemical assays showed that cancer cells selectively activate key glycolytic enzymes and novel ER-stress markers, while protein synthesis is severely suppressed. Interestingly, deprivation of oxygen affected the expression of various epigenetic regulators such as histone demethylases and NuRD (nucleosome remodeling and deacetylase) complex in A431 cells. In addition, we identified PHF14 (the plant homeodomain finger-14) as a novel hypoxia-sensitive epigenetic regulator that plays a key role in cell cycle progress and protein synthesis. Hypoxia-mediated inhibition of PHF14 was associated with increase of key cell cycle inhibitors, p14^ARF^, p15^INK4b^, and p16^INK4a^, which are responsible for G1-S phase transition and decrease of AKT-mTOR-4E-BP1/pS6K signaling pathway, a master regulator of protein synthesis, in response to environmental cues. Analysis of TCGA colon cancer (n=461) and skin cancer (n=470) datasets revealed a positive correlation between PHF14 expression and protein translation initiation factors, eIF4E, eIF4B, and RPS6. Significance of PHF14 gene was further demonstrated by *in vivo* mouse xenograft model using PHF14 KD cell lines.

## INTRODUCTION

Rapidly growing tumor cells that outgrow their vascular supply often encounter oxygen-deficient “hypoxia” condition. Hypoxia, a key characteristic feature of tumor microenvironment, is a pathophysiological stimulus that impacts diverse tumorigenic events such as immunosuppression, angiogenesis, invasiveness, and metastasis, as well as drives malignant transformation and chemoresistance of tumor cells [1–5]. Therefore, decades of cancer research have focused on tumor hypoxia as it triggers oncogenic property of cancer cells and its profound effects on clinical outcome in cancer patients.

Hypoxia upregulates the expression of several pro-angiogenic factors that induce the neo-vascularization of tumor mass [6, 7] and drives metabolic shift from oxidative phosphorylation (OXPHOS) to glycolysis which leads to activation of proteases, resulting in an acidic microenvironment and ultimately, induces tumor metastasis [3, 8]. Although the long term cellular response to hypoxic stress is the triggering of oncogenic potential of cancer cells to escape from oxygen- and nutrient-deficient environment, the immediate response to hypoxia is decreased cell proliferation and cell cycle arrest to reduce excessive oxygen/nutrient consumption in many cancer cell lines [9, 10]. Hypoxia controls cell proliferation and cell cycle mainly via the hypoxia-inducible factor-1α (HIF-1α) signaling axis [10]. HIF-1α, a master regulator of hypoxia [11], has been known to mediate the transcription of cell cycle regulators, such as cyclin-dependent kinase (CDK) inhibitors, p21 and p27, by displacement of c-Myc from its DNA binding site [10, 12]. In addition, HIF-1α has a direct effect on DNA replication machinery and c-Myc protein expression [13, 14]. Through HIF-1α-mediated suppression of c-Myc protein or disruption of c-Myc-Max transcription factor complex, c-Myc-dependent transcription of target genes such as cyclin D2 and E2F1 (the target of retinoblastoma protein) are interrupted [15].

Since hypoxic tumor microenvironment is a main causal factor of malignant tumor progression and poor therapeutic outcome of cancer patients, we hypothesized that tumor cells actively control protein translational events that are critical to their adaptive responses for clonal evolution and tumorigenesis despite under oxygen- and serum-deprivation conditions. In order to investigate the *de novo* protein translation in the early hypoxia response, we employed quantitative pulsed stable isotope labeling with amino acids in cell culture (pSILAC) method to discriminate the newly synthesized proteins from pre-existing ones before hypoxia stress [16] and directly quantify protein translation events of A431 squamous carcinoma cells in response to hypoxia or serum starvation. Study of *de novo* synthesized or translationally suppressed proteins under environmental stress revealed key molecules responsible for metabolic shift, malignant transformation, or epigenetic regulation in cancer cells. More importantly, our approach has discovered a novel pathway of hypoxia-driven cell cycle arrest via epigenetic regulation. We identified PHF14 (the plant homeodomain (PHD) finger-14) as a novel key cell cycle regulator. PHF14, a relatively understudied epigenetic reader, was initially identified as a histone-binding protein through PHD finger motif [17–19]. In this report, we investigated the association between PHF14 and cell cycle arrest in cancer cells. By genetic depletion of PHF14 protein, hypoxic cancer cells increased the expression of CDK inhibitors, p15^INK4b^ and p16^INK4a^, and p53-dependent cell cycle regulator, p14^ARF^, and consequently inhibited G1-to-S phase transition [20, 21]. In addition, PHF14 knockdown was associated with inhibition of AKT-mTOR-4E-BP1/S6K phosphorylation, which implicated that hypoxia-mediated suppression of PHF14 may regulate protein synthesis through AKT-mTOR signaling pathway.

## RESULTS

### Quantitative proteomic analysis of hypoxia-responsive proteins using pSILAC method

**Figure 1:**
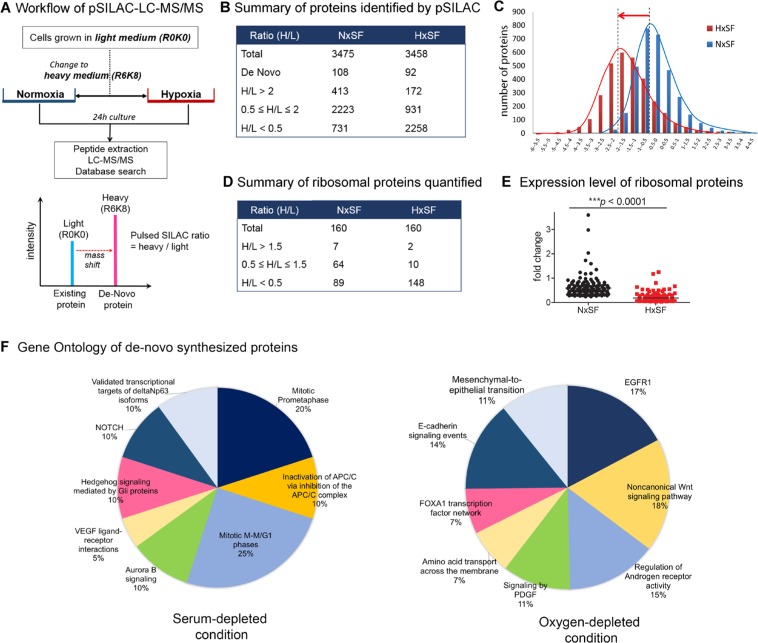
Quantitative pSILAC based proteomic analysis of A431 cells **(A)** Protein labeling and evaluation scheme for pSILAC-LC-MS. A431 cells grown in “light medium” (L, R0K0) were transferred to “heavy medium” (H, R6K8) and cultured for 24 hr under either normoxia or hypoxia. Pre-existing protein was fully labeled R0K0 and newly synthesized protein was labeled R6K8. Protein synthesis ratio was determined by heavy/light labeled peptide. **(B)** Summary of proteins identified by pSILAC-LC-MS/MS in A431 cells under normoxia (NxSF) or hypoxia (HxSF). **(C)** Distribution of protein synthesis ratio (log_2_[H/L]). It clearly indicated the suppression of protein synthesis under hypoxia. **(D)** Summary of ribosomal proteins identified by pSILAC-LC-MS. **(E)** Cellular protein synthesis ratio of ribosomal proteins under normoxia or hypoxia (*p<0.0001*). **(F)** Gene ontology analysis of actively translating proteins in either serum- or oxygen-depleted condition.

To investigate the early cellular response to hypoxic stress, we employed pSILAC-based quantitative proteomic approach to detect synthesis of *de novo* proteins and translational dynamics. The workflow for pSILAC labeling scheme and proteomic analysis is described in Figure 1A and the “Materials and Methods” section. Briefly, A431 cells grown in “light” medium, containing unlabeled [^12^C6, ^14^N2]-Lys and [^12^C6]-Arg, were switched to “heavy” medium, containing labeled [^13^C6, ^15^N2]-Lys and [^13^C6]-Arg for 24 hr. The incorporation of the stable isotopes labeled heavy lysine and arginine in the proteins allowed us to differentiate newly synthesized proteins from pre-existing proteins (Figure 1A). Proteome profiles were acquired from two biological replicates and further analyzed to select target protein groups. The Spearman's rank correlation coefficients between two biological replicates from normoxic or hypoxic cell proteomes were respectively 0.883 and 0.853, confirming a high reproducibility of dataset (Supplementary Figure 1). Key regulated proteins were selected when they appeared in both dataset and further validated by RT-qPCR or western blot analysis to confirm their expression changes.

Comparative analysis of *de novo* synthesized proteins [heavy/light (H/L) ratios] between normoxic and hypoxic A431 cells are presented in Figure 1B and 1C. A total of 3475 proteins and 3452 proteins were identified with at least two unique tryptic peptides using Proteome Discoverer v2.2 (Thermo Fisher Scientific Inc., Waltham, MA, USA) from A431 cells grown under either normoxia or hypoxia for 24 hr in heavy medium, respectively (Supplementary Data 1 and 2). Normoxic A431 cells (NxSF) sustained their protein synthesis without significant induction or reduction even under serum deficient condition while only 11.9% (413 proteins, H/L>2) or 21.0% (731 proteins, H/L<0.5) of total proteins were translationally induced or suppressed, respectively. In contrast, about 65.4% of total proteins were translationally suppressed in hypoxic A431 cells (HxSF) while only 172 proteins (4.9%, H/L>2) were upregulated. It reflects that oxygen deprivation causes a significant widespread suppression of protein synthesis in cancer cells while limited nutrition supply (serum free medium) does not induce an immediate change in protein translation. Since ribosome is an essential cellular machinery of protein synthesis, we next quantified expression levels of ribosomal proteins (Figure 1D, Supplementary Data 3). Of the 160 ribosomal proteins identified in normoxia, 55.6% of identified proteins were downregulated while 44.3% of total proteins were unchanged or slightly upregulated under serum starvation condition. On the contrary, 148 out of 160 ribosomal proteins (91.9%) were suppressed to minimum translation level under hypoxia, reflecting the impairment of protein synthesis machinery under hypoxia (Figure 1E). We further analyzed proteins those exclusively labeled in either normoxia or hypoxia by gene ontology approach using FunRich functional enrichment analysis tool [22]. We found that a set of 98 proteins or 64 proteins were actively translated in serum starvation or low oxygen condition, respectively (Figure 1F, Supplementary Data 4 and 5). Interestingly, PDGF, EGFR and Notch signaling pathway associated proteins were actively translated in hypoxic A431 cells while serum-deprived A431 cells increased VEGF signaling and cell cycle related proteins.

### Hypoxia triggers shift of energy metabolism to glycolysis in cancer cells

**Figure 2:**
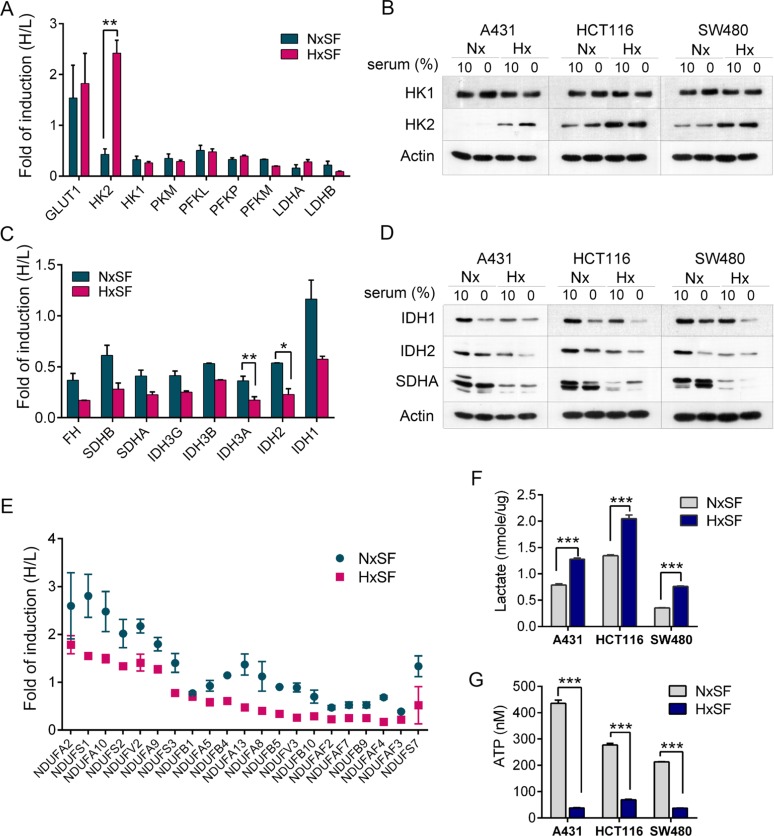
The oxygen and nutrients supplies modulate the expression of key metabolic enzymes in cancer cells **(A)** Protein synthesis ratio of key glycolytic enzymes. Expression of GLUT1 and HK2 was increased in hypoxic A431 cells (HxSF). Induction fold was normalized by pre-existing proteins. Error bars represent the mean±s.d., n=2. ^**^p<0.01. **(B)** A431, HCT116, or SW480 cells were cultured at the indicated condition for 24 hr and subjected to western blot analysis for HK1 and HK2. Actin was used as loading control. **(C)** Protein synthesis of key TCA cycle enzymes was suppressed in A431 cells grown in serum-depleted condition. Induction fold was normalized by pre-existing proteins. Error bars represent the mean±s.d., n=2. ^**^p<0.01, ^*^p<0.05. **(D)** A431, HCT116, or SW480 cells were cultured at the indicated condition for 24 hr and subjected to western blot analysis for key TCA cycle enzymes. Actin was used as loading control. **(E)** Hypoxia suppressed protein synthesis of mitochondrial complex I NADH dehydrogenase (NADH:ubiquinone oxidoreductase core subunits, NDUFs) in A431 cells. Induction fold was normalized by pre-existing proteins. **(F)** Three hypoxic cancer cells, A431, HCT116, and SW480, displayed more than a 1.5-2 fold increase in lactate levels compared with normoxic cells. Error bars represent the mean±s.d., n=3. ^***^p<0.001 **(G)** Three hypoxic cancer cells, A431, HCT116, and SW480, displayed a significant reduction in intracellular ATP levels compared with normoxic cells. ATP contents (nM) were compared in equal number of cells. Error bars represent the mean ± s.d., n=3. ^***^p<0.001. Abbreviations: Nx, normoxia; Hx, hypoxia; SF, serum free.

Cancer cells utilize aerobic glycolysis to generate the energy required for cellular process instead of mitochondrial OXPHOS [23, 24]. Analysis of glycolytic enzymes in pSILAC data revealed that glucose transporter 1 (GLUT1) and hexokinase 2 (HK2), key mediators of aerobic glycolysis [25, 26], were *de novo* synthesized more than 1.8-fold higher upon hypoxic stress while most glycolytic enzymes including HK1, pyruvate kinase muscle isozyme (PKM), Phosphofructokinase isozyme PFKL (liver), PFKP (platelet), PKFM (muscle), lactate dehydrogenase A (LDHA), and LDHB were suppressed (Figure 2A) [25, 26]. Interestingly, HK2 was exclusively responsive to hypoxia but not to serum deprivation (*p=0.0092*) while *de novo* protein synthesis of GLUT1 was induced upon serum starvation regardless of oxygen supply, indicating that HK2 is a key mediator of hypoxia-induced glycolysis. Induction of HK2 was further validated by western blot analysis in different cancer cell lines, SW480 and HCT116 (Figure 2B).

Metabolic alterations in cancer cells are tightly linked with mitochondrial dysfunctions that inhibit OXPHOS [27]. pSILAC data showed that mitochondrial tricarboxylic acid (TCA) metabolic enzymes that primarily generate NADH and FADH_2_ for OXPHOS were suppressed under both serum-depleted normoxia and hypoxia conditions. As shown in Figure 2C and 2D, translation of key TCA enzymes including isocitrate dehydrogenase 2 (IDH2), IDH3A, IDH3B, IDH3G, succinate dehydrogenase complex A (SDHA), SDHB, and fumarate hydratase (FH), was suppressed upon serum deprivation regardless of oxygen level, indicating that serum plays a major role in determining protein synthesis of TCA enzymes. In line with this observation, protein synthesis of mitochondrial complex I NADH dehydrogenases, regulators of NAD+/NADH ratio that ultimately lead to energy production [24], tended to be downregulated although serum depletion showed the modest effects than hypoxia (Figure 2E). To further confirm the effects of mitochondrial dysfunction and enhanced glycolysis on cellular metabolism in response to hypoxia, lactate and ATP levels were measured in A431, SW480, and HCT116 cells. As shown in Figure 2F and 2G, lactate levels were elevated while ATP levels were significantly decreased in three hypoxic cancer cells. Collectively, cancer cells adapt to hypoxic microenvironment by modulating a key glycolytic enzyme, HK2, while limited nutrient supply causes wide range of metabolic stress in mitochondria regardless of oxygen supply, implying that shift of energy metabolism to glycolysis of cancer cells is mainly initiated by hypoxia.

### pSILAC data reveals primary hypoxia responsible proteins in cancer cells

**Figure 3:**
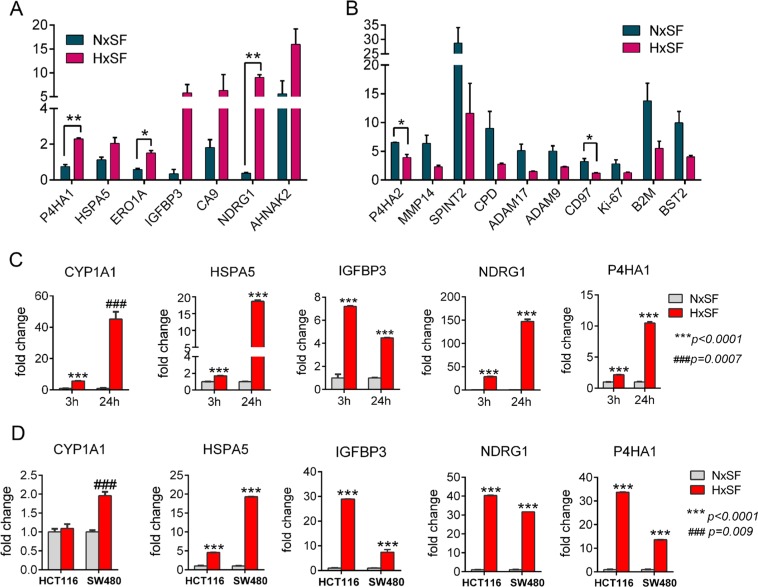
Quantitative pSILAC data reveals primary hypoxia responsible proteins in cancer cells **(A)** Hypoxia induced protein synthesis of oxidative stress markers and ER stress response proteins in A431 cells grown in serum free medium. Induction fold was normalized by pre-existing proteins. Error bars represent the mean±s.d., n=2. ^**^p<0.01, ^*^p<0.05. **(B)** Serum depletion induced protein synthesis of angiogenesis related proteases and cancer biomarkers in A431 cells. Induction fold was normalized by pre-existing proteins. Error bars represent the mean±s.d., n=2. ^*^p<0.05. **(C–D)** qRT-PCR analysis of 5 selected genes in A431, HCT116, and SW480 cells showed increase of mRNA expression under hypoxia. Error bars represent the mean ± s.d., n=3. ^***^p<0.001. Abbreviations: Nx, normoxia; Hx, hypoxia; SF, serum free.

Cancer cells suppress *de novo* synthesis of numerous proteins to reduce energy consumption during hypoxic condition. However, a set of 172 proteins which are actively synthesized in the unfavorable hypoxia environment are likely to play key roles in cellular adaptation mechanism. Since newly synthesized proteins can take a primary role for cellular resistance or adaptation mechanism to hypoxia, we first analyzed hypoxia-mediated highly upregulated proteins. Quantitative pSILAC data showed that hypoxic cancer cells highly increased translation of oxidative stress marker proteins while most other protein translations were suppressed. As expected, hypoxia marker proteins such as carbonic anhydrase 9 (CA9) and N-Myc downstream regulated 1 (NDRG1) [28, 29] were increased about 6-to-9 fold compared to pre-existing proteins within 24 hr after exposure to hypoxia (Figure 3A). In addition, endoplasmic reticulum (ER) stress response proteins such as 78kDa glucose-regulated protein (HSPA5) [30] and endoplasmic reticulum oxidoreductase alpha (ERO1L) [31] were upregulated around 2-fold whereas maintained relatively low levels under normoxia (Figure 3A). Prolyl 4-hydroxylase subunit alpha (P4HA1), a key enzyme in collagen synthesis [32] and insulin growth factor binding protein 3 (IGFBP3) [33] were also detected as highly synthesized proteins compared to normoxia control (Figure 3A). Cytochrome P4501A1 (CYP1A1), an important player of initiation of carcinogenesis [34], was exclusively upregulated as compared to other hypoxia marker proteins under the influence of hypoxia. Interestingly, several angiogenesis-related proteases such as matrix metalloproteinase 14 (MMP14), carboxypeptidase D (CPD), ADAM9, and ADAM17 (disintegrin and metalloproteinase domain-containing protein 9 and 17) [35–37] and cancer biomarkers including CD97 (cluster of differentiation 97), BST2 (bone marrow stromal antigen 2), B2M (beta-2 microglobulin), and Ki-67 [38–41] were rapidly synthesized in response to serum-starvation but not to oxygen-depleted condition, indicating that low serum stress alone could promote tumor angiogenesis and contribute to the development of cancer-specific markers (Figure 3B). RT-qPCR data was provided in Supplementary Figure 2.

To validate the expression level of those newly synthesized proteins, we selected a cluster of upregulated proteins or exclusively expressed protein in hypoxia such as NDRG1, HSPA5, IGFBP3, P4HA1, and CYP1A1 for RT-qPCR analysis at different time points (3 hr and 24 hr). In consistent with our pSILAC results, expression level of CYP1A1, NDRG1, HSPA5, P4HA1, and IGFBP3 transcripts was significantly upregulated after 24 hr of hypoxia culture and this tendency was detectable as early as 3 hr time point (Figure 3C). pSILAC and RT-qPCR results of 5 selected genes in A431 cells were further examined in different cancer cells, HCT116 and SW480. Overall, RT-qPCR results of these genes in HCT116 and SW480 cells were in line with A431 cells’ results except CYP1A1 gene (Figure 3D). Especially, NDRG1 and P4HA1 showed significant induction of gene transcription in the three hypoxic cells compared to their respective normoxic controls.

### Hypoxia modulates protein synthesis of epigenetic regulators in cancer cells

**Figure 4:**
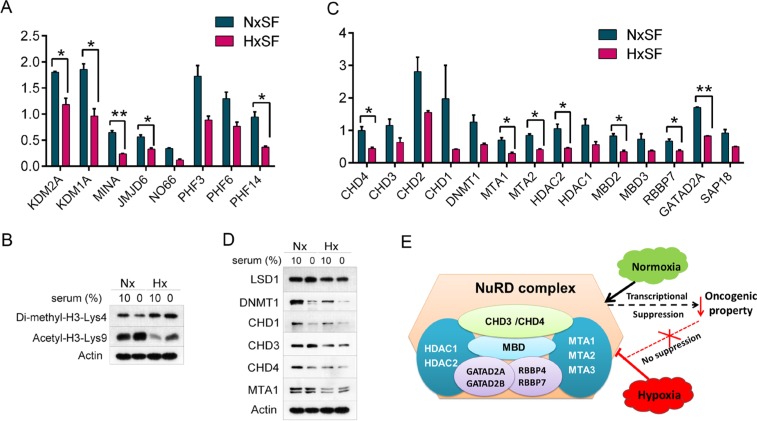
Availability of oxygen and nutrients modulates expression of epigenetic regulators in cancer cells **(A)** Hypoxia inhibited protein synthesis of histone demethylases in A431 cells. Induction fold was normalized by pre-existing proteins. Error bars represent the mean±s.d., n=2. ^**^p<0.01, ^*^p<0.05. **(B)** Western blot analysis of histone modification indicated that histone methylation level was increased while acetylation level was decreased under hypoxia regardless of serum supply. **(C)** Hypoxia suppressed protein synthesis of NuRD complex in A431 cells. Induction fold was normalized by pre-existing proteins. Error bars represent the mean±s.d., n=2. ^**^p<0.01, ^*^p<0.05. **(D)** NuRD protein levels, assessed by western blot analysis, indicated that either hypoxia or hypo-nutrients condition suppressed the expression of NuRD complex in A431 cells. Actin was used as loading control. **(E)** Role of NuRD complex as transcriptional corepressor on oncogenic factors that can revert back by hypoxia-mediated suppression of NuRD complex. Nx, normoxia; Hx, hypoxia; SF, serum free.

Recent progress in understanding of cancer metabolism reveals that hypoxia-driven metabolic changes affect epigenetic regulation of cancer cells. Several TCA cycle intermediates such as NAD^+^, citrate, and α-ketoglutarate, can be exported out of mitochondria and provided as substrates or cofactors of epigenetic regulators which are directly associated with gene expression [42, 43]. Since pSILAC data showed dysregulation of glycolysis/TCA cycle enzymes and suppression of protein translation under reduced oxygen/nutrients availability, we examined if translation of epigenetic regulators such as DNA or histone modifiers accounting for gene transcription were changed. pSILAC proteomic data, acquired from two independent biological replicates, showed suppression of key epigenetic regulators upon hypoxia. As shown in Figure 4A, protein synthesis of Jumonji C (JmjC) domain-containing proteins which possess histone demethylase activity was decreased in both serum- and oxygen-depleted conditions. These included MINA and NO66, bifunctional histone lysine-specific demethylases and histidyl-hydroxylases, and JMJD6 (jumonji domain-containing protein 6), a histone arginine demethylase [43–45]. In contrast, normoxic A431 cells grown in serum depleted medium enhanced protein synthesis of lysine specific demethylase 1A (KDM1A), KDM2A, PHD finger protein 3 (PHF3) and PHF6 while translation of these demethylases was suppressed when A431 cells exposed to hypoxia, indicating the modulatory role of hypoxia on histone-mediated epigenetic regulation [46]. It was further verified by western blot analysis for histone modification status (Figure 4B). In concordance with pSILAC data, methylation level of histone H3 lysine 4 (H3K4me2) was increased upon decrease of corresponding histone demethylases, MINA and NO66.

Further screening of DNA or histone modifying enzymes revealed an inhibition of NuRD (nucleosome remodeling and histone deacetylase) complex in hypoxic A431 cells (Figure 4C). Under hypoxic condition, A431 cells showed about 60 – 70% reduction in *de novo* synthesis of NuRD complex compared to pre-existing proteins including HDAC2 (histone deacetylases 2), CHDs (chromodomain helicase DNA binding protein), MBDs (methyl-CpG-binding domain protein), MTAs (metastasis-associated gene), and GATA 2A/2B (GATA binding protein 2A/2B). Expression level of NuRD complex was further validated by western blot analysis (Figure 4D). NuRD complex collaborates with various recruiting partners in cancer progression as transcriptional corepressor or coactivator [47]. Tumor suppression was mediated by recruitment of NuRD complex with SALL1 and by releasing of this complex from chromatin, cancer cells can be rescued from cell senescence [48]. In addition, NuRD complex with NAB2, a co-repressor of the early growth response (EGR) family of transcriptional transactivator, repress EGR activities that promote progression of prostate cancer [49]. Collectively, inhibition of NuRD complex under hypoxia can release cancer cells from suppressive effects on oncogenic potentials such as Snail, TGFβ signaling, focal adhesion process, or MAPK activities (Figure 4E) [47].

### Identification of PHF14 as a key cell cycle regulator under hypoxia

**Figure 5:**
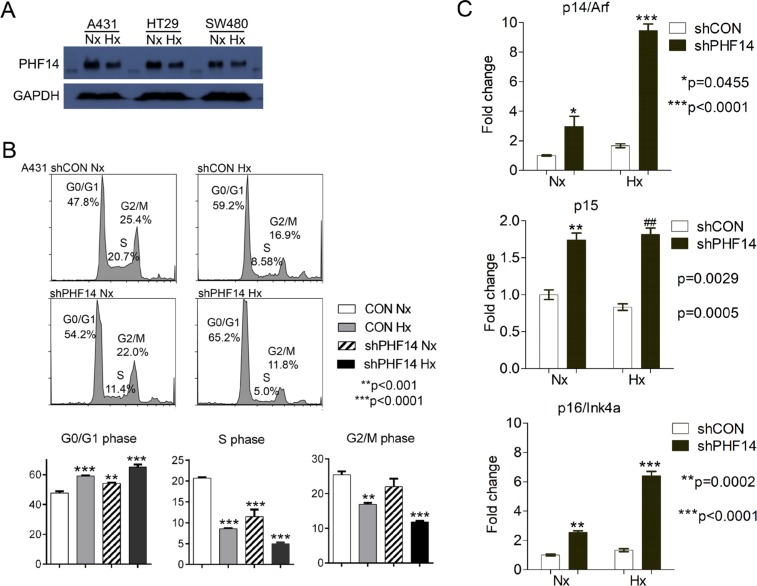
Depletion of PHF14 inhibits G1-to-S phase transition **(A)** Expression of PHF14 was determined by western blot analysis in three cancer cell lines. Note that PHF14 expression was decreased in hypoxic cancer cells. GAPDH was used as loading control. Nx, normoxia, Hx, hypoxia. **(B)** Flow cytometry analysis of PI-stained A431-con and A431-shPHF14 cells. Data indicated that PHF14 knockdown inhibited G1-S transition compared with control and suppression level was enhanced when A431-shPHF14 cells were grown in hypoxia. **(C)** Depletion of PHF14 induced the expression of cell cycle inhibitors, p14^ARF^, p15^INK4b^, and p16^INK4a^.

Quantitative pSILAC analysis of early response proteins under hypoxia revealed a key cell cycle regulator in A431 cells. We found that protein synthesis of PHD finger protein 14 (PHF14), a relatively unknown histone binding protein [17], was suppressed under hypoxia while PHF3 and PHF6, another members of PHD finger protein family, maintained their expression level (Figure 4A). Suppression of PHF14 in hypoxic condition was further confirmed by western blot analysis in A431, SW480, and HT29 cells (Figure 5A).

To investigate if PHF14 is functionally associated with hypoxia-mediated cellular response, we first analyzed the cell cycle arrest, a common feature of hypoxia, using A431 cells stably expressing non-target shRNA (A431 con) or shRNA against PHF14 (A431-shPHF14) for protein depletion (Figure 5B).

Compared with corresponding control, A431 PHF14 knockdown cells induced G0/G1 cell cycle arrest and S-phase inhibition (Figure 5B) similar to hypoxia treatment. Specifically, PHF14 knockdown cells showed 50% reduction in S-phase compared to control, indicating that PHF14-mediated cell cycle regulation is related with G-to-S phase transition. To further characterize the role of PHF14 in cell cycle progression, we examined the expression of key cell cycle regulators for G1/S arrest such as p15^INK4b^ and p16^INK4a^, cyclin-dependent kinase inhibitors, and p14^ARF^, a p53-dependent cell cycle inhibitor. As shown in Figure 5C, expression of p15^INK4b^ and p16^INK4a^ was induced in PHF14 knockdown cells and induction level was much higher in hypoxic A431-shPHF14 cells. Interestingly, expression of p14^ARF^, an alternatively spliced gene of the INK4b-ARF-INK4a locus, was increased in both normoxic and hypoxic PHF14 knockdown A431 cells. Genes resulting from alternative reading frame products of INK4b-ARF-INK4a locus are known to be tumor suppressor genes and induce cell cycle arrest. In many cancer cells, these genes are controlled by epigenetic tools such as DNA methylation or histone demethylation, indicating the potential role of PHF14 as an epigenetic regulator in early response stage of cells to hypoxic stress.

### PHF14 modulates AKT-mTOR signaling pathway in cancer

PHF14 has a histone binding motif that potentially regulates the expression of target genes by modulating histone. So far little is known about the exact function and target of PHF14 in gene transcription. In this study, we found that several key cell cycle regulators were transcriptionally modulated by PHF14 (Figure 5C). Hence, we further characterized the effects of PHF14 on signaling pathways that integrate a variety of external stimuli to control cell growth and proliferation.

**Figure 6:**
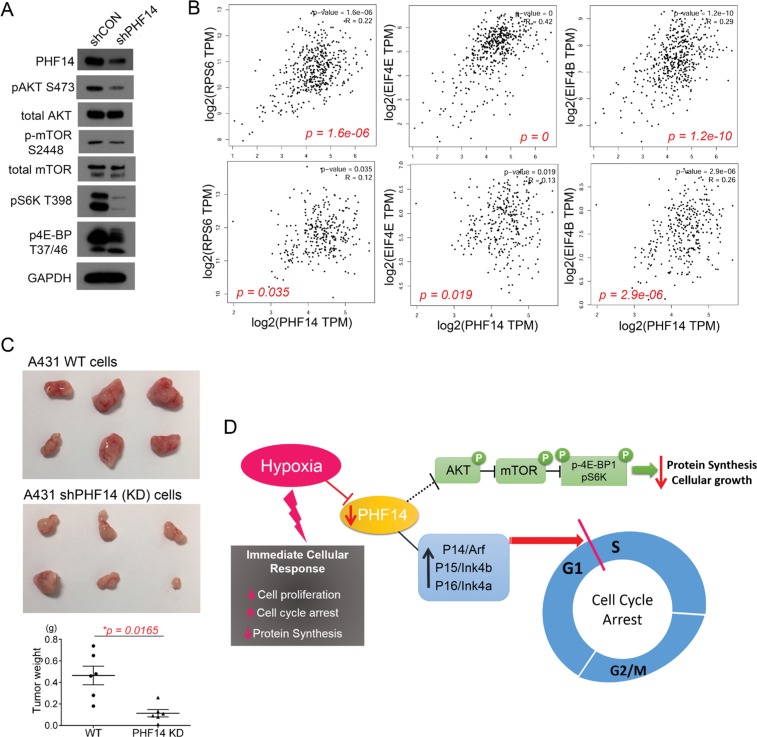
PHF-14 correlates diverse signaling pathway in A431 cells **(A)** Depletion of PHF14 inhibited AKT-mTOR-4E-BP1/S6K pathway, a master regulator of protein synthesis. **(B)** Correlation between PHF14 gene expression and RPS6, EIF4E, or EIF4B expression in TCGA-SKCM (upper panel) and TCGA-COAD (lower panel). **(C)** PHF14-mediated cell cycle inhibition was further assessed in *in vivo* mouse xenograft model. A431-con or A431-shPHF14 cells were subcutaneously injected to the flank of recipient nude mice. Tumor burden was measured by assessment of tumor gross weight. Statistical differences between groups were determined using Student's t-test (mean ± s.d., n = 6/group, ^*^p = 0.0165). **(D)** Proposed role of PHF14 under hypoxia. Hypoxia-mediated suppression of PHF14 was associated with cell cycle arrest and inhibition of protein synthesis.

It is well known that mTOR (the mechanistic target of rapamycin) regulates protein synthesis upon nutrient or oxygen availability via modulation of substrate phosphorylation such as eukaryotic translation initiation factor 4E (eIF4E)-binding protein 1 (4E-BP1) and p70 S6 kinase (p70S6K) [50, 51]. Since PHF14 was suppressed under hypoxic stress, we first analyzed whether suppression of PHF14 was related with phosphorylation status of mTOR and its upstream or downstream effectors, AKT, 4E-BP1, or p70S6K. As shown in Figure 6A, depletion of PHF14 reduced the phosphorylation level of AKT S473, mTOR S2448, 4E-BP1 T37/46, and p70S6K T389. It implies that suppression of mTOR signaling pathway, a master regulator of protein synthesis responding to environmental cues, is modulated by PHF14 expression under hypoxia. To further investigate a correlation between PHF14 gene expression and cellular protein synthesis, we analyzed expression of PHF14 gene and key regulator of protein synthesis such as ribosomal protein S6 (rpS6), eukaryotic initiation factor 4E (eIF4E), or eukaryotic initiation factor 4B (eIF4B) in the Cancer Genome Atlas (TCGA) dataset [52], TCGA-SKCM (skin cutaneous melanoma, n=470) and TCGA-COAD (colon adenocarcinoma, n = 461), using GEPIA platform [53]. Analysis of two cancer datasets clearly indicated that gene expression of PHF14 was positively correlated with expression of those eukaryotic translation initiation factors (Figure 6B). Therefore, expression level of PHF14 is associated with status of protein translation in cancer cells.

To examine if *in vitro* inhibition role of PHF14 on cell cycle progression and protein synthesis was also reflected in an *in vivo* model, we performed subcutaneous (s.c.) injection of A431 con or A431 PHF14 KD cells into the flank of NCr nude mice (n=6 per group). We observed a significant inhibition in growth of tumors derived from the PHF14 KD cells compared with controls (p = 0.0165; Figure 6C). The mean weight of tumors generated by A431 PHF14 KD cells was 63.6% of that generated by A431-con cells assessed at the same time point, indicating that depletion of PHF14 can potently restrict tumor growth *in vivo*. Figure 6D shows a proposed model of PHF14 under hypoxic condition. As shown in this study, hypoxia-mediated suppression PHF14 is closely correlated with general features of cellular responses against hypoxia, such as cell cycle arrest and inhibition of protein synthesis.

## DISCUSSION

Hypoxia, a hallmark of the tumor microenvironment, is a pathophysiological cue that is associated with multiple tumorigenic processes such as immunosuppression, angiogenesis, invasion, metastasis, metabolic reprogramming, autophagy induction, drug resistance, and poor clinical outcomes [1, 2]. When facing hypoxic stress, cells initiate several adaptive responses such as cell cycle arrest to reduce excessive oxygen consumption and activate pro-angiogenic or survival factors [7, 9]. These events are regulated by various cellular pathways including the HIF-1α-mediated gene regulation, mTOR signaling, and autophagy activation [3, 5, 50, 51].

The aim of this study is to investigate the early cellular response mechanism under hypoxia and to unveil the primary modulators at the early stage of hypoxic stress through quantitative pSILAC-based proteomic analysis. Analysis of total proteome from normoxic or hypoxic A431 cancer cells clearly elucidated the cellular adaptation mechanism to hypoxia.

Firstly, hypoxia induces selective induction and suppression of hypoxia-associated proteins in cancer cells. Hypoxic A431 cells increased de novo synthesis of hypoxia inducible proteins (only 4.9% of total proteome) that are required for cell survival under limited oxygen and growth factors in serum-free conditions while translation of most cellular proteins, especially ribosomal proteins, was severely inhibited. It includes ER stress response proteins such as HSPA5 and ERO1L, and oxidative stress marker proteins such as IGFBP3, CA9, and NDRG1.

Secondly, A431 cells suppress de novo synthesis of epigenetic modulators such as histone demethylases or deacetylases including MINA, NO66, JMJD6, and NuRD complex within 24 hr after exposure to hypoxic stress. Among these modulators, NuRD complex has received much attention because of a contradictory role in tumor progress as transcriptional coactivator or corepressor with various binding partners. Thus, our findings of adverse effects of hypoxia on NuRD complex may provide another clue to reveal the hypoxia-inducible binding partners of NuRD complex. Since epigenetic changes are directly linked to various gene expressions, hypoxia-mediated modulation of epigenetic regulators could be an efficient way to induce a phenotypic shift in cancer cells.

Lastly, analysis for epigenetic modulators under hypoxia has led to identification of PHF14 as a key cell cycle regulator. Hypoxic treatment was able to suppress the expression of PHF14 at transcriptional and translational levels in A431 cells, suggesting that PHF14 could be an early response protein under hypoxia. Study for correlation between hypoxia-mediated cellular response and PHF14 expression showed that PHF14 knockdown inhibited G1-to-S phase transition by upregulation of CDK inhibitors, p15^INK4b^ and p16^INK4a^, and a p53-dependent cell cycle inhibitor, p14^ARF^. In addition, PHF14 was involved in AKT-mTOR-4E-BP1 signaling pathway and expression level of PHF14 was positively correlated with several key translation factors in TCGA skin and colon cancer patient dataset, indicated that hypoxia-mediated suppression of PHF14 gene is related with cellular protein synthesis under stress condition. While expression of PHF14 was directly related with mRNA level of CDK inhibitors, AKT-mTOR signaling pathway was regulated by phosphorylation cascade, indicating that PHF14 may contribute to the expression of upstream ligands as an epigenetic regulator. Expression of PHF14 was appeared to be correlated in several cancer models such as biliary tract cancer (BTC), colon cancer, and lung cancer [19, 54, 55]. PHF14 has been reported as a potential diagnostic marker of lung cancer as its overexpression is inversely correlated with carcinogenesis and poor survival. PHF14 can promote tumorigenesis through increasing cell proliferation and DNA instability after forming a functional complex with KIF4A. In line with our findings, depletion of PHF14 suppressed cell proliferation in several lung cancer cells although its respective role was associated with mitotic defect not G1-S phase arrest. On the contrary, earlier finding in BTC cells reported that knocking down PHF14 gene enhanced BTC cell growth. Generally epigenetic modifiers regulate gene expression by the assembly of relevant interacting partners, thus, contradict outcome of PHF14 depletion in different cancer cells might be associated with its functional binding partners and environmental stress may affect the complex formation. Hence, epigenetic role of PHF14 on possible targets needs to be further investigated. While a potential role of PHF14 in cancer was studied in context of epigenetic regulator or transcription factor, little is still known about upstream regulators of PHF14. Recently, it was appeared in a possible correlation between DHHC-type protein acyltransferase, DHHC3 and PHF14 as depletion of DHHC down-regulated PHF14 in cancer but underlying mechanism remains obscure [56].

Collectively, PHF14, a chromosome-binding epigenetic reader, can target diverse cellular signaling pathways to modulate fundamental cellular functions, especially cell growth and cell cycle regulation responding to low oxygen. Inhibition of PHF14 expression can significantly constrain energy consuming process such as cell cycle progression and protein synthesis. Our findings have thus unveiled the novel exploitable role of hypoxia-sensitive PHF14 by cancer cells to adapt to the stressful oxygen-depleted tumor microenvironment.

## MATERIALS AND METHODS

### Cell culture and treatments

Human squamous carcinoma cells (A431) and human colon cancer cell lines (HCT116, HT29 and SW480) were purchased from the American Type Culture Collection (Manassas, VA, USA). Cells were cultured in RPMI-1640 or DMEM medium supplemented with 10% fetal bovine serum (FBS; HyClone; GE Healthcare Life Sciences, Logan, UT, USA) and 1% (v/v) penicillin/streptomycin in a humidified incubator at 37˚C with 5% CO_2_. For serum depletion (serum-free; SF) experiments, cells were washed twice in PBS and fresh medium without FBS was added, supplemented with 1% (v/v) penicillin/streptomycin and cultured for 24 hr. For hypoxia experiments, cells were placed into a hypoxic chamber containing a gas mixture of 95% N_2_ and 5% CO_2_.for 24 hr. A shRNA used for knockdown of PHF14 expression was designed and purchased from OriGene Technologies, Inc (TG310460, Rockville, MD, USA). Scrambled shRNA was used as a control. A431 cells stably expressing non-target shRNA or PHF14-targeting shRNA constructs were generated by puromycin selection for 2 weeks.

### Western blot analysis

Cells were lysed using modified RIPA buffer (50 mM Tris–HCl, 150 mM NaCl, 1% NP-40, pH 8.0, 1 × protease inhibitor cocktail) and lysates were subjected to western blotting with the indicated primary antibodies. Antibodies to HK1, HK2, Di-methyl-H3-Lys4, Acetyl-H3-Lys9, LSD1, DNMT1, CHD1, CHD3, CHD4, MTA1, AKT, pAKTS473, mTOR, p-mTOR S2448, pS6K T398, p4E-BP T37/46, and HDAC1, 2, 3, 6 were purchased from cell signaling technologies (Beverly, MA, USA). Antibodies to IDH1, IDH2, and SDHA were from GeneTex, Inc. (Irvine, CA, USA). Anti-PHF14 antibody was from Proteintech Group, Inc. (Rosemont, IL, USA). Actin and GAPDH antibodies were obtained from Millipore (Billerica, MA, USA). Proteins bound by these antibodies were detected using a chemiluminescent detection kit (Pierce; Thermo Fisher Scientific, Inc., Waltham, MA, USA).

### Pulsed-SILAC experiments

Two independent biological replicates were performed. A431 cells grown in “light” medium, containing unlabeled 146 mg/l ^12^C_6_, ^14^N_2_-L-lysine and 84 mg/L ^12^C_6_-L-arginine, were switched to “heavy” medium, containing labeled 146 mg/l ^13^C_6_, ^15^N_2_-D-Lys and 84 mg/l ^13^C_6_-D-Arg, and then cultured in normoxic or hypoxic conditions for 24 hr. Cells were washed with cold PBS, lysed with 8 M urea buffer containing a protease inhibitor cocktail, and in-solution digestion was performed as previously described [57]. Extracted peptides were subjected to fractionation on an Xbridge™ C18 column (4.6 × 250.0 mm; Waters Corporation, Milford, MA, USA) and subsequent analysis by liquid chromatography-tandem mass spectrometry (LC-MS/MS).

Peptides were separated and analyzed on a Dionex Ultimate 3000 RSLCnano system coupled to a Q Exactive instrument (Thermo Fisher Scientific, Inc.). Separation was performed on a Dionex EASY-Spray 75 μm x 10 cm column packed with PepMap C18 3μm, 100 Å (Thermo Fisher Scientific, Inc.) using solvent A (0.1% formic acid in 5% I) and solvent B (0.1% formic acid in 90% ACN) at flow rate of 300 nl/min with a 60 min gradient. Peptides were then analyzed on a Q Exactive apparatus with an EASY nanospray source (Thermo Fisher Scientific, Inc.) at an electrospray potential of 1.5 kV. A full MS scan (350-1,600 m/z range) was acquired at a resolution of 70,000 at m/z 200 and a maximum ion accumulation time of 100 ms. Dynamic exclusion was set as 15 s. The resolution of the higher energy collisional dissociation (HCD) spectra was set to 17,500 at m/z 200. The automatic gain control settings of the full MS and MS2 scans were 3E6 and 2E5, respectively. The 10 most intense ions above the 2,000-count threshold were selected for HCD-fragmentation, with a maximum ion accumulation time of 100 ms. An isolation width of 2 was used for MS2. Single and unassigned charged ions were excluded from MS/MS. For HCD, the normalized collision energy was set to 28%. The underfill ratio was defined as 0.2%.

Raw data files from the three technical replicates were processed and searched using Proteome Discoverer 2.2 (Thermo Fisher, MA, USA) together with the Sequest and Mascot search engines. A standard search type with 2 multiplicity, 3 maximum labelled amino acids and heavy labelled Lys6 and Arg8 were used for pSILAC quantitation. The first and main searches for peptide mass tolerance were 20 and 4.5 parts per million (ppm.), respectively, while the MS/MS match tolerance was 20 p.p.m. with Fourier transform mass spectrometry de-isotoping enabled. The absence of two trypsin cleavage sites per protein was allowed. Carbamidomethylation (C) was set as a fixed modification. Oxidation (M) and deamidation (NQ) were set as variable modifications. The search was performed in Revert decoy mode with peptide spectrum match false discovery rate (FDR), protein FDR and site decoy fraction set to 0.01. The FDR for protein identification was <1%, and fold-change log2 value >|1.0| was defined as differential expression.

### RNA extraction and RT-qPCR

RNA extraction was performed using Nucleospin RNA kits (MACHEREY-NAGEL GmbH & Co. KG, Düren, Germany) according to the manufacturer's protocol. Quantitative real-time PCR was performed using the CFX96 Real-Time PCR Detection System (Bio-Rad Laboratories, Inc., Hercules, CA, USA) with the KAPA SYBR^®^ FAST qPCR Master Mix. Actin or 18sRNA were used as internal controls. The primer sequences used for qPCR are provided in the supplementary information (Supplementary Table 6).

### DNA content analysis using propidium iodide (PI) staining

Flow cytometric analysis of PI-stained cells was performed to demonstrate the effects of hypoxia and PHF14 depletion on cell cycle progression. Briefly, cells were harvested at the indicated time points, washed twice with ice-cold PBS and fixed in 70% ethanol overnight at 4˚C. Before flow cytometry, cells were washed twice with ice-cold PBS and stained with 1 ml PI (15 μg/ml; Sigma-Aldrich; Merck KGaA, Darmstadt, Germany) containing 2.5 μg/ml RNase A (Roche Diagnostics, Basel, Switzerland) for at least 30 min. The DNA content of at least 10,000 cells was determined using a BD LSR II flow cytometer (Becton Dickinson, San Jose, CA, USA). The proportion of cells in a particular phase of the cell cycle was determined using BD FACSDIVA software (Becton Dickinson).

### Mouse xenograft model

All animal studies were approved by an Institutional Animal Care and Use Committee (IACUC; ARF-SBS/NIE-A0242) and were performed in accordance with approved guidelines and regulations of the Animal Facility Center of the School of Biological Sciences, Nanyang Technological University, Singapore. NCr nude mice (8 weeks, male) were obtained from InVivos Pte., Ltd. (Singapore). The mice received subcutaneous injection of 1 × 10^6^ A431-con (n=6) or A431-shPHF14 (n=6) cancer cells into either the left or right flank, and tumor size was monitored with a caliper. When the tumors reached 1.5 cm in diameter, the mice were euthanized as per the Animal Facility Center guidelines.

### Statistical analysis

Statistical analysis was performed using SPSS 18.0 statistical software (SPSS, Inc., Chicago, IL, USA). Statistical differences between variables were determined using Student's paired t-test, and P<0.05 was considered to represent a statistically significant difference. Correlation between PHF14 expression and RPS6, eIF4E, or eIF4B expression was analyzed via GEPIA platform [53] and the Pearson correlation coefficient in TCGA-SKCM (Skin Cutaneous Melanoma) and TCGA-COAD (Colon adenocarcinoma) dataset. Cancer patient datasets used in this research are generated by the TCGA Research Network: http://cancergenome.nih.gov/.

## SUPPLEMENTARY MATERIALS FIGURES AND TABLES












