# Supplementary material for: Pulsed SILAC-based proteomic analysis unveils hypoxia- and serum starvation-induced de novo protein synthesis with PHD finger protein 14 (PHF14) as a hypoxia sensitive epigenetic regulator in cell cycle progression
Source: Oncotarget. 2019 Mar 15;10(22):2136–50. doi: 10.18632/oncotarget.26669 (PMC6481330; doi:10.18632/oncotarget.26669)
Supplement: Supplementary file 1 [file oncotarget-10-2136-s001.pdf]

Pulsed SILAC-based proteomic analysis unveils hypoxia- and serum starvation-induced *de novo* protein synthesis with PHD finger protein 14 (PHF14) as a hypoxia sensitive epigenetic regulator in cell cycle progression

SUPPLEMENTARY MATERIALS

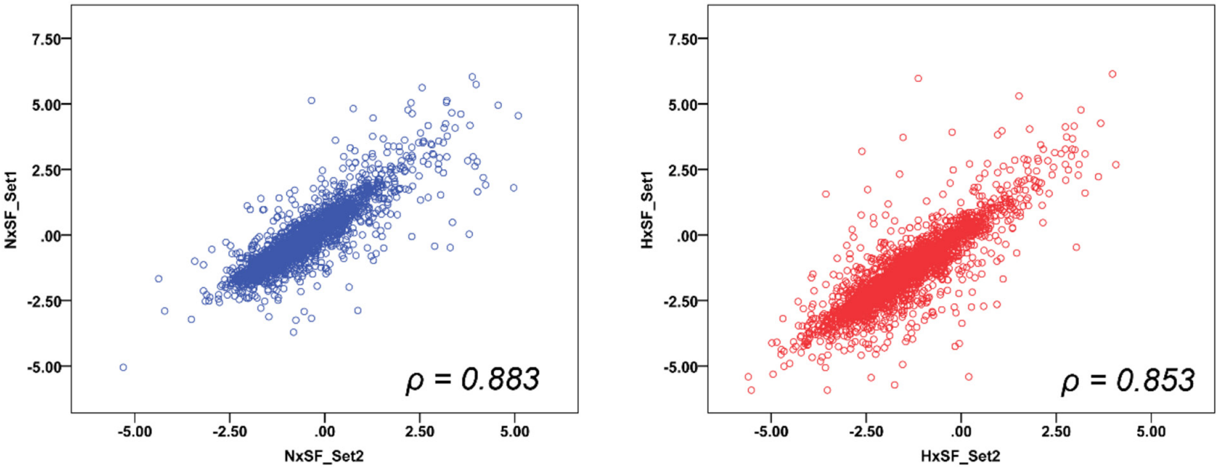

Supplementary Figure 1: The Spearman's rank correlation coefficients between two biological replicates from normoxic or hypoxic cell proteomes.

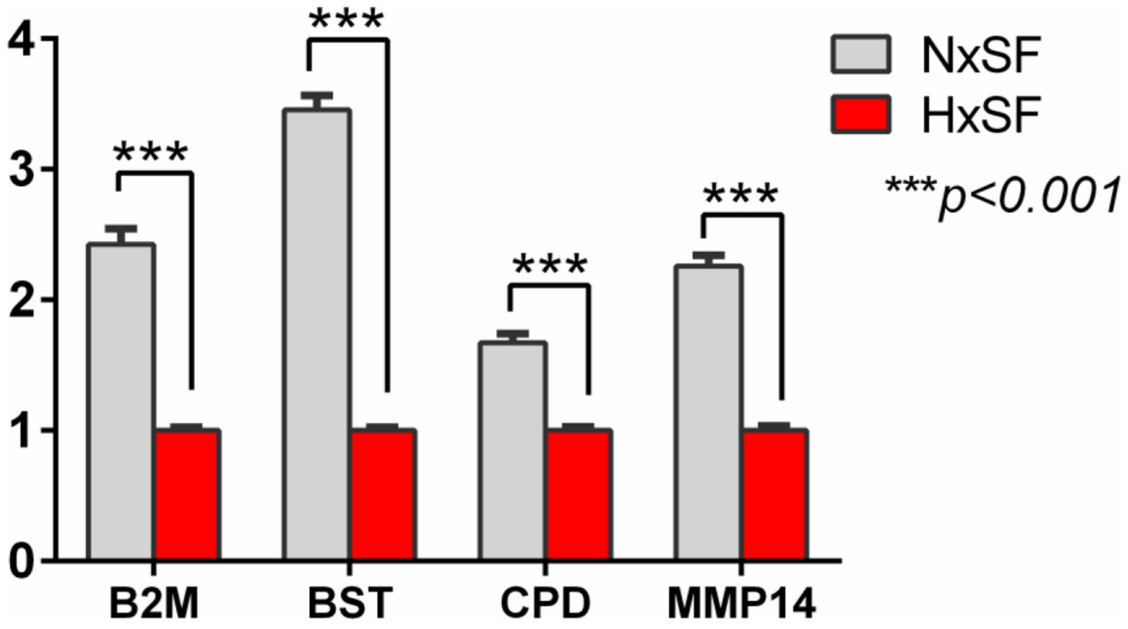

Supplementary Figure 2: qRT-PCR analysis of Angiogenesis-related proteases and cancer biomarkers in A431 cells. Error bars represent the mean±s.d., n=3. ****p<0.001*. Nx, normoxia; Hx, hypoxia; SF, serum free.

Supplementary Table 1: Primer Sequences for the quantitative PCR

Primer	5' - 3'
**link to Figure 3 Hypoxia Responsible proteins**	
IGFBP3 F	CCTGCCGTAAGAAAATGGAA
IGFBP3 R	AGGCTGCCCATACTTATCCA
CRELD2 F	CTGCTCTCCAGGAACCTACG
CRELD2 R	CCTGACTGTGCAGATGCTGT
CYP1A1 F	CTTGGACCTCTTTGGAGCTG
CYP1A1 R	CGAAGGAAGAGTGTCGGAAG
HSPA5 F	GGTGAAAGACCCCTGACAAA
HSPA5 R	GTCAGGCGATTGTCGTCATT
NDRG1 F	ACAACCCTGAGATGGTGGAG
NDRG1 R	TGTGGACCACTTCCACGTTA
P4HA1 F	GGCAGCCAAAGCTCTGTTAC
P4HA1 R	GGCTTGTTCCATCCACAGTT
B2M F	GGG CCT CAG TGA TTC ACA TT
B2M R	AGG GAG ACC AAG GGA TGA TT
BST2 F	TGC TGG GGA TAG GAA TTC TG
BST2 R	TCA GCT CTT GTT GCA GGA GA
MMP14 F	CAC TGC CTA CGA GAG GAA GG
MMP14 R	TTG GGG TAC TCG CTA TCC AC
CPD F	GAG TGA AGG CGC TAT TCA GG
CPD R	TGA GGC TTT CCA AAC CAT TC
**link to Figure 5 Cell Cycle Inhibitors**	
p14ARF F	GAAAGGCCCTCGAAAAGTCG
p14ARF R	GGCGGTTATCTCCTCCTCCTA
p15INK4b F	GGGGGTCGGGTAGAGGA
p15INK4b R	GCGCTGCCCATCATCAT
p16INK4a F	AGCCGCCCACAACGACTTTATTTT
p16INK4a R	GTTATTCCCGGTCGGCTCCTCCTT

Supplementary Data 1:

See Supplementary File 1

Supplementary Data 2:

See Supplementary File 2

Supplementary Data 3:

See Supplementary File 3

Supplementary Data 4:

See Supplementary File 4

Supplementary Data 5:

See Supplementary File 5
